# Does human-induced hybridization have long-term genetic effects? Empirical testing with domesticated, wild and hybridized fish populations

**DOI:** 10.1111/eva.12199

**Published:** 2014-08-27

**Authors:** Andrew Harbicht, Chris C Wilson, Dylan J Fraser

**Affiliations:** 1 Department of Biology, Concordia UniversityMontreal, QC, Canada; 2 Aquatic Biodiversity and Conservation Unit, Ontario Ministry of Natural ResourcesPeterborough, ON, Canada

**Keywords:** adaptive potential, domestic, hatchery, hybridization, phenotypic plasticity, *Salvelinus fontinalis*, selection, survival, transplant

## Abstract

Current conservation practices exclude human-generated hybridized populations from protection, as the genetic effects of hybridization in the wild have been observed to be long-lasting based on neutral genetic markers and are considered potentially irreversible. Theory, however, predicts otherwise for genes under selection. We transplanted combinations of wild, domesticated and hybridized populations of a fish species to new environments. We then compared survival, phenotypic variation and plasticity to determine whether hybridization affects adaptive potential after multiple generations of selection in the wild. Although the fitness of our hybridized populations at the onset of hybridization cannot be assessed, our results suggest that within five to eleven generations, selection can remove introduced foreign genes from wild populations that have hybridized with domesticated conspecifics. The end result is hybridized populations that, in terms of survival, phenotypic plasticity, mean trait expression and overall general responses to environmental change, closely resemble neighbouring wild populations. These results have important implications for considering the potential conservation value of hybridized populations and illustrate the effectiveness of selection in a local environment.

## Introduction

Human-induced hybridization is increasing worldwide, often with unpredictable outcomes with respect to species persistence (Tallmon et al. 2004). Such hybridization is generally seen as a problem (Rhymer and Simberloff 1996), with researchers focusing thus far on its immediate fitness consequences in the first couple of generations (e.g. McGinnity et al. 2003; Araki et al. 2007) and its possible long-term neutral genetic effects (Hansen 2002). Very few studies, however, have evaluated the long-term effects on fitness in nature (but see Johnson et al. 2010), a significant gap for conservation. For example, current regulations often exempt human-induced hybridized populations from the protection given to pure wild populations, under the assumption that because of their altered ‘genetic integrity’, the hybridized populations might continue to carry maladaptive traits (e.g. Allendorf et al. 2004; COSEWIC 2011). Some theory, however, predicts that this may not be the case (Edmands 1999). More empirical research is needed to assess the conservation value of already hybridized populations and to decide how best to consider them in biodiversity conservation (Allendorf et al. 2001), species restoration (Hansen and Mensberg 2009) and fisheries/wildlife management (Araki et al. 2007).

Intraspecific hybridization is common among exploited fish species, particularly salmonids (Utter 2000; Fleming and Petersson 2001; Hansen et al. 2009; Fraser et al. 2010). Although salmonid populations are often adapted to their local environments (Fraser et al. 2011), their high socio-economic importance has led to widespread stocking of hatchery/domesticated conspecifics into wild populations to compensate for population declines resulting from habitat alteration or fishing pressure (Aprahamian et al. 2003). Such stocking practices expose wild populations to hatchery strains that routinely originate from other regions and/or that have experienced intentional or unintentional selection, typically resulting in reduced fitness in the wild in hatchery fish, and F1 or F2 hatchery-wild hybrids (Araki et al. 2007; Fraser 2008).

Due to a number of factors, the long-term effect of fitness reductions in hatchery-wild hybrids is usually unknown. For example, if hybrid fitness depends primarily on strong interactions between individual genotypes and the environment, then natural selection should remove foreign, maladaptive alleles quickly, returning populations to their previous state after a relatively short period of time (Edmands 2007). Yet introduced maladaptive alleles may persist over the long term if the immigration rate of hatchery fish is high, if selection is weak or if selection is only strong episodically, such as during floods, forest fires and droughts (Allendorf et al. 2004; Hansen et al. 2009). Furthermore, population size may affect hybrid fitness and the duration of effects. Maladaptive alleles may become fixed via genetic drift in populations that were small prior to hybridization (Rieseberg et al. 1999; Ellstrand and Schierenbeck 2006). But in certain situations, small wild populations might also benefit from the introduction of domestic/hatchery alleles if these mask deleterious recessive alleles that are common within inbred populations (Fraser et al. 2010). Large populations may also benefit from some hybridization when experiencing environmental change; the increased genetic variation generated may increase the capacity to respond to new selective pressures (Swindell and Bouzat 2006).

Overall, despite considerable levels of hybridization in many cases and concerns over possible long-term genetic effects, hatchery-wild hybridized populations often persist after stocking has ceased at what are considered ‘normal’ densities (Halbisen and Wilson 2009; Hansen and Mensberg 2009). Such an observation might be viewed as evidence that selection removes maladaptive alleles following hybridization, returning population fitness to levels more like closely related nonstocked populations. Whether this actually occurs is seldom-tested because of considerable logistical challenges. Indeed, to do so requires making a comparison of the performance of the pre- and posthybridization state of a population after natural selection has acted for multiple generations.

Alternatively, one can contrast the performance of hatchery, hybridized and closely related nonhybridized populations when exposed to novel environmental change, namely transplantation to new environments. Hybridized populations that maintain introduced, potentially beneficial alleles over the long term (for reasons discussed above) may exhibit increased adaptive potential in the form of greater survival and phenotypic plasticity, as a result of elevated genetic diversity (Arnold 1992; Dowling and Secor 1997). Conversely, hybridized populations that have had maladaptive introduced alleles removed by natural selection should not have elevated genetic diversity and should exhibit similar capacities to respond to new environments when compared to closely related, nonstocked populations, having been reverted to a more wild-like state. Lastly, where selection on maladaptive gene complexes in hybridized populations is weak, fitness might not have returned to prehybridization levels. Under this scenario, the benefit of elevated genetic diversity provided to hybridized populations might be offset by the presence of maladaptive genes, resulting in the intermediate performance of hybridized populations between nonhybridized and hatchery populations.

To distinguish between these predictions, we performed matched, experimental transplants of hatchery, hybridized and wild (nonhybridized) brook trout (*Salvelinus fontinalis*) populations from Algonquin Park, Ontario, Canada into three new environments. Algonquin populations have a long and well-documented stocking history that has produced many hybridized populations with varying levels of mixing (Harbicht et al. 2014). Stocking of a nonlocal hatchery strain ceased within the park for naturally self-sustaining brook trout populations in 1989 with very few exceptions, so many park populations now represent hybridized populations exposed to natural and artificial (angling) selection for at least five to eleven generations (Blanchfield et al. 2003). Many park populations were also excluded from stocking and represent closely related wild populations against which hybridized populations can be compared.

Our predictions made two main assumptions: (i) alleles introduced into wild populations following hybridization with hatchery fish can be beneficial in new environments and (ii) hatchery-wild hybrid fitness is always lower than wild fish in the local environments of wild fish. We therefore complemented our experimental transplants by performing a meta-analysis that compared the survival of domesticated-wild salmonid hybrids relative to pure wild fish in nature. If such a meta-analysis showed that domesticated-wild hybrids outperform wild fish in new environments, this would suggest that domesticated alleles provide a fitness advantage to hybridized populations in such environments. Secondarily, if domesticated-wild hybrid fitness is always lower than wild fish within the local environment of wild fish, this was likely to be true for within our Algonquin study area. It would therefore help to discern between the aforementioned predictions, namely that of reversion to wild-like states by natural selection.

## Methods

### Source populations

**Figure 1:**
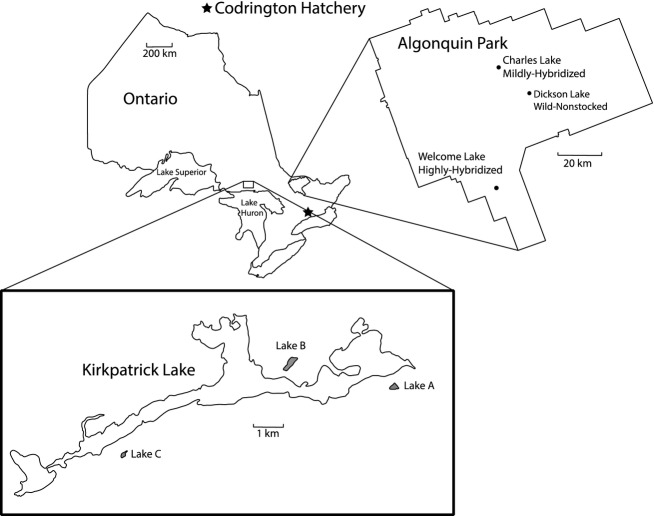
A map outlining the geographic locations of the three Algonquin Park source populations used as gametic sources, the location of the hatchery where source population crosses were incubated, as well as the three experimental transplant lakes.

The four transplanted populations (Table 1) originated either from the hatchery or from Algonquin Park in Ontario, Canada, and spanned a range of exposure to hatchery fish, hereafter referred to as the hatchery, wild-nonstocked, mildly hybridized and highly hybridized populations (Fig. 1). The hatchery population (the Hills Lake strain) is maintained at a high effective population size within the Ontario hatchery system and is used for stocking throughout the province. The wild-nonstocked population (Dickson Lake) was never stocked with hatchery fish. The hybridized populations (mildly hybridized = Charles Lake; highly hybridized = Welcome Lake) were both stocked previously (as recently as 1994 and 1978, respectively; Table 1). Harbicht et al. (2014) found that both populations were admixed with hatchery genes (most individuals possess some genetic material originating from the Hills Lake hatchery strain), with the extent of introgression being estimated at 18% (mildly hybridized population) and 69% (highly hybridized population).

**Table 1:** Environmental characteristics of source population habitat and experimental lakes used in a transplant experiment as well as information on the mean percentage of hatchery admixture (i.e. nonadmixed = 0) based on Harbicht et al. (2014)

Population/Lake	Surface area (ha)	Mean depth (m)	Secchi depth (m)	Shoreline development index	Species Present (predators/total)	Stocking history (first, last, no. of events)	Total number of hatchery fish stocked
Wild-Nonstocked	974.7	16.8	5.6	2.91	4/14	NA	NA
Mildly Hybridized	12.3	3.4	6.4	1.93	1/5	1954, 1994, 5	4700
Highly Hybridized	469.7	7.53	4.13	1.70	0/9	1940, 1978, 10	47 226
Hatchery	NA	NA	NA	NA	NA	NA	NA
Lake A	3.9	4.0	2.5	1.36	0/1	NA	NA
Lake B	2.5	6.4	5.0	1.46	0/1	NA	NA
Lake C	8.7	3.3	5.5	1.49	0/0	NA	NA

Population/Lake	Hatchery admixture	Full/Half sibling families	Spawning date (yy, mm, dd)
Wild-nonstocked	0	12/0	10, 11, 03
Mildly hybridized	18	15/2	10, 11, 02
Highly hybridized	69	11/2	10, 11, (03,10)
Hatchery	100	16/0	10, 11, (01,16)
Lake A	NA	NA	NA
Lake B	NA	NA	NA
Lake C	NA	NA	NA

### Experimental crosses

Male and female gametes were collected and combined in Algonquin Park between the 1st and 3rd of November 2010 with the exception of two families from the hatchery strain and four families from the highly hybridized population that were added later to boost family numbers. A total of eleven to sixteen full-sibling families were created for each population (Table 1). Exceptions to full-sibling families were the result of insufficient females available at the time of gamete collection, in which case two half-sibling families were created from a single female. Fertilized eggs were then incubated at Codrington Hatchery, 180 km south of Algonquin Park. All families were initially kept separate from one another while experiencing identical conditions (water source, feeding schedule, temperatures). Just prior to yolk sac absorption (approximately 110–115 days after fertilization), families were combined according to their population of origin, and feeding began. Shortly thereafter (121–130 days after fertilization), fish were measured, and densities among populations were equalized.

### Transplants, field sampling and survival in new environments

Forty-one days prior to transplanting, fry from all populations were combined in equal proportions into three different holding tanks corresponding to the three transplant lakes 380 km northwest of Algonquin Park: Lake A (Penikett Lake), Lake B (Woodside Lake) and Lake C (Roy Berry Lake) (Fig. 1). Mortality among the source populations following pooling was minimal and did not exceed 5 fish per study lake postpooling. The densities stocked were constant among lakes based on a stocking ratio of 1000 fry/ha. The three transplant lakes are all situated in the Penokean Hills on the North Shore region of Lake Huron, Ontario, an area undergoing considerable postglacial isostatic rebound (Sella et al. 2007) which has made recolonization by fish species difficult. This, combined with the possibility of periodic winterkill, has resulted in lakes A and B containing only one minnow species (*Notropis* sp.), while Lake C is fishless. Transplants were conducted using a helicopter on 15 May 2011 (180–195 days after fertilization). Due to maternal differences in egg size investment, a difference in fry size was present prior to stocking, with fry from the mildly hybridized population being significantly larger than the similarly aged fry from the other three populations (Appendix S1).

The three transplant lakes were revisited 5 months after planting in October 2011. Fish were captured using short gill net sets (from 15 min to 7 h; soak times increased towards the end of the study to increase catches) with two 182.8 cm × 27.4 m gill nets consisting of three equally sized panels of 1.27, 1.9 and 2.54 cm stretched monofilament mesh, capable of capturing all possible size classes of fish present in this study. Captured fish were placed into a recovery pail and left for 15 min prior to being anesthetized with MS222 (Tricaine methanesulfonate). Anesthetized fish were then weighed, measured and photographed for phenotypic analyses below using an overhead mounted Nikon D40 digital SLR camera (Nikon Corporation, Chiyoda, Tokyo, Japan). Adipose fins were then removed and stored in 95% ethanol before fish were released back into the lake. Details of subsequent DNA extractions and genotyping at fourteen polymorphic microsatellite loci (including PCR and gel electrophoresis conditions) are found in Harbicht et al. (2014).

Adipose fin clips were also used to identify previously captured trout for a mark–recapture analysis of abundance. To estimate abundances within each transplant lake, the FSA package within R (Ogle 2011) was implemented. This involved using the Schnabel method with the Chapman modification, adopted when the proportion of the total population caught per capture event is <0.1 and the proportion of the population that is marked is <0.1 (Chapman 1954). A Poisson distribution was used to construct confidence limits as the total number of recaptured fish never exceeded 50 for any of the lakes throughout the sampling period (Krebs 1999).

To compare survival among source populations, each captured (and genotyped) trout was first assigned to its known parents (and hence its source population), based on Mendelian exclusion methods implemented in the SOLOMON package in R (Christie et al. 2013). Of the recaptured fish, 90% assigned back to a single parental pair, and another 9% assigned back to multiple families within the same source population (25 of the 36 such fish were from the mildly hybridized population which had low genetic diversity). The few individuals that could not be assigned back to a single source population were excluded from further analyses. Mismatching alleles were permitted to account for possible genotyping error; the vast majority of offspring (89%) assigned back to a single parental pair after allowing three alleles to mismatch. Survival was then compared among source populations using the numbers of assigned fish per net set (capture event) in a generalized linear model (GLM). The data were significantly overdispersed (*P*-value < 0.001), so a quasi-Poisson error distribution was used. Models included the source population and the study lake as explanatory variables and were compared using quasi-AIC (Burnham and Anderson 2002).

A second analysis on survival was conducted to ensure that differences were the result of genetics and not the extent of environmental change experienced by each source population when transplanted to a new environment. This was accomplished by modelling catches against their corresponding habitat dissimilarity index (HDI) created using the procedure of Cheong (1992). The HDI included information on the surface area (ha), mean depth (m), Secchi depth (m) and shoreline development index of each lake, and hence, the hatchery population was omitted from this supplemental analysis.

The use of catch per capture event as a proxy for survival assumed that any variation in soak times and differences in the number of capture events did not introduce bias. Supplemental analyses indicated that there were no significant relationships to suggest bias in the catches towards one source population or another (Appendix S2).

### Phenotypic trait expression in new environments

We compared variation in size and body morphology among transplanted fish as measures of the phenotypic means and variation in new environments. To measure the extent of variation, photos of the left side of each fish were first uploaded into the program TPSDIG2 (Rohlf 2006). Seventeen landmarks were then placed (Appendix S3) on each photo to be analysed in TPSRELW (Rohlf 2006). A consensus body shape (generalized orthogonal least-squares Procrustes mean) was constructed using the mean landmark positions corrected for angle, scale and centroid size. The program then aligned each sample to the consensus using thin-plate spline analyses (Bookstein 1989), and from this, a partial warp analysis (Bookstein 1991) was performed. Such an analysis returns two-dimensional relative warp (RW) values that represent the direction and magnitude of deviations from the consensus form. A single consensus shape and partial warp analysis was performed using photos of trout captured in all three lakes and combined into a single data set. Trout that deviated from the standard salmonid form due to damage suffered in gill nets were omitted from this analysis.

Centroid size (the cumulative radial distances from a central position to each landmark) was used as a measure of body size due to the highly significant linear relationship between centroid size and fork length (*R*^2^ = 0.99, *P* ≪ 0.01) and exponential relationship with mass (*R*^2^ = 0.97, *P* ≪ 0.01). The morphological variation explained by the RWs trailed off noticeably following RW4; therefore, no RWs beyond that were considered in the analysis. RW1, RW3 and RW4 accounted for 28.5%, 9.7% and 7.7% of the total body form variation, respectively. Higher values of these relative warps corresponded roughly to increased abdominal body depth (RW1), increased body depth to body length ratio (thickness) (RW3) and lengthened caudal peduncles (RW4). RW2 (14.6% of the total variation) corresponded to the extent of bending of the spine and was only predominant among fish that were photographed post-mortem. It was concluded that this was likely the result of rigour mortis, and therefore, RW2 was excluded from further analysis.

Phenotypic plasticity was contrasted between and within study lakes. Firstly, RW values and centroid sizes were modelled in GLMs to test for significant interactions between source populations and study lakes. Significant evidence of an interaction would indicate that slopes, and therefore reaction norms across environments, differed among populations. Additive and interactive models for RW1, RW3 and RW4 as well as centroid size were constructed and compared using AIC values. Centroid size was included as an explanatory variable when comparing RW values to account for effects of allometric growth. Secondly, the homogeneity of variation around the means of RW1, RW3, RW4 and the centroid size for the four source populations was compared within each study lake using a Fligner–Killeen test. This allowed us to test for elevated levels of plasticity in the hybridized populations as proposed by Arnold (1992).

### Meta-analysis

We meta-analytically compared the survival of domesticated-wild hybrids and wild fish across salmonid studies conducted in nature, by firstly conducting keyword searches in ISI Web of Science™ (Thompson Reuters Corporation, New York, NY, USA) and Google Scholar™ (Google, Mountain View, CA, USA) using combinations of the following keywords: hybrid, hatchery, wild, population, survival and fitness. References within studies were also screened to find relevant articles not obtained through keyword searches.

We chose survival as the relative fitness component to compare because it is commonly measured and easily standardized. It also corresponded closely with our study's scope. Only studies in nature meeting the following criteria were retained for analyses: (i) the wild component in hybrids was from the same population as the wild fish to which hybrids were compared; (ii) wild and hybrid fish experienced the same environmental rearing conditions; and (iii) the wild population had no history of being stocked with hatchery fish prior to fitness comparisons (*sensu* recommendations of Fraser 2008). We also recorded whether hybrid and wild fish survival was compared in the local environment of the wild population or a foreign one.

We then calculated the effect size of the relative proportion of recaptured wild and hybrid fish using the log odds ratio (Lipsey and Wilson 2001). Data were standardized using the following equation:


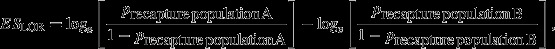


where *ES*_LOR_ is the effect size of the log odds ratio, *p*_recapture population A_ is the proportion of recaptured wild fish and *p*_recapture population B_ is the proportion of recaptured domesticated-wild hybrid fish. Thus, a positive effect size indicates that wild fish survive better than hybrids, the converse for a negative effect size. For each effect size, we calculated an error term (*SE*_LOR_) using the following equation:


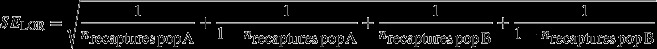


The weights (*w*_LOR_) associated with each effect size estimate were then calculated using:


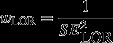


We used *ES*_LOR_ as the dependent variable in linear models, including *w*_LOR_ to weight each effect size based on its sample size. We began with a full model including, as explanatory variables, (i) whether the domesticated strain used to create hybrids was a local or nonlocal strain, (ii) whether the testing environment was the wild population's local environment or a foreign one, (iii) the life stage at stocking and (iv) the maximum duration of exposure to natural conditions. An exhaustive model search was then performed using maximum likelihood to fit the models to the various combinations of fixed effects and comparing model fit using both AIC comparisons (Akaike 1974) and log likelihood ratio tests.

## Results

### Transplant population abundances and survival

Abundance estimates for the three transplant lakes were similar and had strongly overlapping 95% confidence intervals: 568 (348–984), 567 (322–1076) and 392 (160–967). Trout transplanted into all three lakes experienced similarly high mortalities during the study period ranging from 80% (Lake C) to 96% (Lake B).

**Figure 2:**
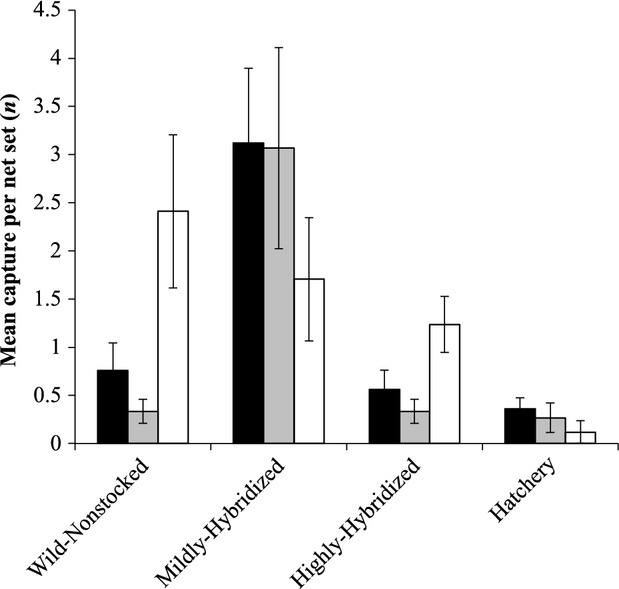
Mean (±SE) number of fish caught per net set from each of four transplanted populations introduced to three lakes 380 km from their home environments. 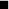, Lake A; 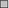, Lake B; 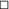, Lake C.

The best-fit model for explaining variance in the number of fish caught, and hence survival, was an interactive model including source population and study lake (Table 2). In all three study lakes, the wild-nonstocked population did not differ significantly from the highly hybridized population in terms of catch numbers, while that of the mildly hybridized population exceeded the wild population significantly in two of three lakes (lakes A and B). The two hybridized populations differed significantly from one another in two of three lakes (lakes A and B). Catches of the mildly hybridized population significantly exceeded those of the hatchery population in all three lakes, while the highly hybridized population significantly exceeded the hatchery population in one of the three study lakes (Fig. 2; Appendix S4). A secondary analysis including habitat dissimilarity found no significant correlation with survival.

**Table 2:** Model selection results for the number of fish caught per capture event using generalized linear models and a quasi-Poisson error distribution

Variable	Parameters (*K*)	Log likelihood	QAIC	ΔQAIC
Study lake × Source population	3	−342.98	297.70	0.00
Source population	1	−370.80	303.70	6.04
Study lake + Source population	2	−368.98	306.30	8.60
Intercept model	0	−447.04	358.10	60.43
Study lake	1	−445.22	360.70	62.99

### Phenotypic trait expression

**Figure 3:**
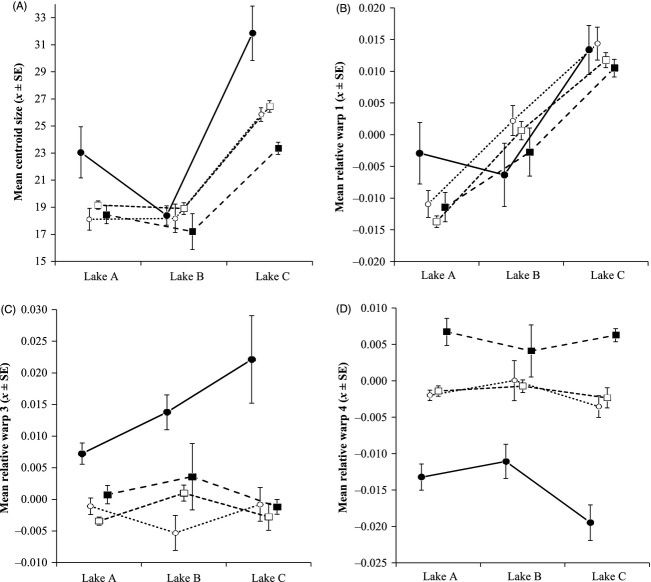
Mean (±SE) values for the centroid sizes (A) and relative warps: RW1 (B), RW3 (C), RW4 (D) for four populations of brook trout in three transplant lakes 380 km from their home environments, calculated using tpsRelW by Rohlf (2006) and photographs of the left side of captured fish. 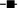, wild-nonstocked; 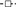, mildly-hybridized; 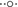, highly-hybridized; 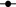, hatchery.

In two of three lakes, the wild-nonstocked and the hybridized populations had strongly overlapping body sizes (centroid sizes), differing significantly only in one lake (Lake C, Fig. 3A; Appendix S5). The hatchery population was significantly larger than all other populations in lakes A and C, but did not differ in Lake B (Fig. 3A; Appendix S5).

For morphological traits, there was little appreciable difference between the wild and hybridized populations in all three study lakes as they all expressed similar abdominal body depths, body thickness and caudal peduncle lengths. The only chief exception was the wild-nonstocked population that had longer caudal peduncles (greater RW4 value) than both hybridized populations in lakes A and C while expressing a thicker body shape (higher RW3 value) than the mildly hybridized population in Lake A (Fig. 3D; Appendix S5). Conversely, in all three study lakes, the hatchery population routinely expressed different body morphologies from the other populations: (i) greater abdominal depth (RW1) in Lake A while displaying reduced abdominal depth in Lake B, (ii) significantly greater overall body thickness in all three lakes (RW3) and (iii) the shortest caudal peduncle length (RW4) in all three study lakes (Fig. 3B–D; Appendix S5).

Body size (centroid) and body morphology variance on RW3 were best described by interactive relationships between source population and study lake (Table 3, Fig. 3). With RW3, however, a simpler additive model was within ΔAIC = 2, so there was little support for differing reaction norms in this case (Burnham and Anderson 2002). Best-fit models for morphological trait variance on RW1 and RW4 were both additive (Table 3, Fig. 3), and morphological trait variance around the mean among source populations within each study lake did not vary significantly in any of the 12 tests (Fligner-Killeen test, all *P* > 0.05).

**Table 3:** Results of model selection using AIC values on the phenotypic parameters (centroid size, relative warp 1, relative warp 3, and relative warp 4) measured using tpsRelW (Rohlf 2006) for four populations of brook trout from Algonquin Park transplanted into three study lakes north of Lake Huron, Ontario, Canada

Response variable	Model	Log likelihood	d.f.	AIC	Δ AIC
Centroid	Lake × Source	−565.99	13	1158.00	0.00
	Lake + Source	−574.08	7	1162.20	4.18
	Lake	−591.43	4	1190.90	32.88
	Source	−667.09	5	1344.20	186.19
	Intercept	−671.40	2	1346.80	188.81
RW1	Lake + Source + Centroid	855.94	8	−1695.90	0.00
	Lake × Source + Centroid	860.79	14	−1693.60	2.31
	Lake + Centroid	848.38	5	−1686.80	9.12
	Lake	795.68	4	−1583.40	112.53
	Lake × Source	804.15	13	−1582.30	113.58
RW3	Lake × Source + Centroid	835.90	14	−1643.80	0.00
	Lake + Source + Centroid	828.99	8	−1642.00	1.83
	Lake × Source	833.68	13	−1641.40	2.45
	Lake + Source	827.62	7	−1641.20	2.57
	Source + Centroid	824.73	6	−1637.50	6.35
RW4	Lake + Source + Centroid	901.51	8	−1787.00	0.00
	Source + Centroid	896.71	6	−1781.40	5.62
	Lake × Source + Centroid	903.09	14	−1778.20	8.86
	Source	885.93	5	−1761.90	25.17
	Lake + Source	887.02	7	−1760.00	26.99

### Meta-analysis

**Figure 4:**
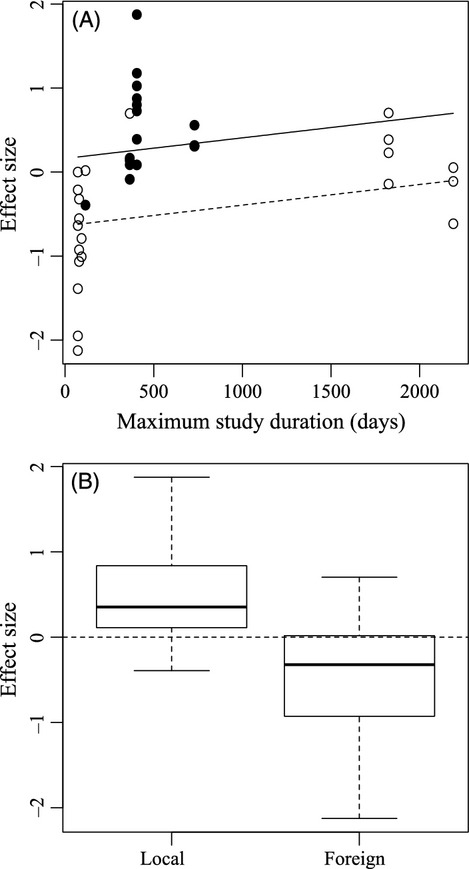
The relationship between effect size and the maximum duration of each survival experiment with the fitted lines according to the status of the local population (A) as well as the mean (±SE) effect sizes against the status of the local wild populations in relation to the experimental environment (local or foreign) (B). 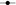, local; 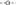, foreign.

Our meta-analysis contained 37 survival comparisons from seven studies in nature between domesticated-wild hybrids and wild fish, that fulfilled criteria for inclusion; sample sizes ranged from 406 to 71 216 individuals stocked and recaptured (mean = 7973) (Appendix S6). Model selection revealed that the best-fit model incorporated whether the study was conducted in the wild population's local environment or a foreign one, and the duration of the study, with each having significant effects (Table 4, Fig. 4). When comparisons were made within the wild population's local environment, the most relevant case to consider from the standpoint of how hybrids initially performed in Algonquin Park lakes, wild fish almost unanimously had higher survival than domesticated-wild hybrids (14 of 16 comparisons, Fig. 4). Conversely, hybrids had better survival than wild fish in foreign environments, the most relevant case for how hybridized populations deal with environmental change (Fig. 4). Study environment (whether local or foreign for the wild population) was also present in both other models within two delta AIC units of the best-fit model (Table 4).

**Table 4:** Meta-analysis best-fit models as determined by an exhaustive model fitting procedure and information theoretic model selection criteria applied to additive models

Domestic status	Study duration	Life-stage stocked	Wild status	d.f.	log likelihood	AIC	ΔAIC
	+		+	4	−31.97	71.90	0.00
	+	+	+	6	−30.54	73.10	1.14
+	+		+	5	−31.85	73.70	1.77
+	+	+	+	7	−30.42	74.80	2.90
			+	3	−35.57	77.10	5.20
+			+	4	−35.54	79.10	7.15
		+	+	5	−34.63	79.30	7.32
+	+			4	−36.17	80.30	8.41
+				3	−37.47	80.90	9.01
+		+	+	6	−34.59	81.20	9.24
+		+		5	−36.41	82.80	10.87
+	+	+		6	−36.09	84.20	12.24
		+		4	−38.36	84.70	12.78
				2	−40.44	84.90	12.93
	+	+		5	−38.28	86.60	14.62
	+			3	−40.42	86.80	14.90

## Discussion

Our results found little evidence supporting that hybridized populations exhibited elevated genetic diversity and concomitant adaptive potential after five to eleven generations of selection in the Algonquin Park environment. While the mildly hybridized population had greater survival than a closely related wild-nonstocked population in two of three new environments, there were no differences in survival between the highly hybridized and wild-nonstocked populations. There also was very little evidence to support that hybridized populations had greater phenotypic variation or plasticity over the long term as has been theorized (Arnold 1992; Tallmon et al. 2004) and observed experimentally over the short term (Swindell and Bouzat 2006; Lucek et al. 2010; but see Morris et al. 2011).

Overall then, the two hybridized and wild-nonstocked populations all had comparable survival, body sizes and body morphologies in new environments, with the exception of higher survival in two lakes for the mildly hybridized population. This result is consistent with the idea that directional selection towards the local environmental optimum in Algonquin Park has occurred within the two hybridized populations (Jordan 1991; Nagy 1997). Indeed, reviewing the phenotypic data (Fig. 3), it is apparent that the wild and hybridized populations more closely resemble each other than the hatchery strain. Meanwhile, only the hatchery strain consistently exhibited both lower survival and strongly divergent phenotypes relative to other populations. This supports previous findings that selection in the hatchery environment reduces the fitness of hatchery fish in the wild (Araki et al. 2007). Alternatively, the poor performance of the hatchery fish may reflect a maternal effect. Although we were unable to verify this through comparisons of egg size, we think it is unlikely the case as a large number of females (16) were used to create the hatchery fish, as well, 41 days prior to stocking the hatchery fry were not significantly different in size relative to both the wild-nonstocked and highly hybridized population fry prior to stocking, yet their survival rates were lower in most cases.

The lack of evidence that introduced hatchery alleles affected the survival, plasticity and the amount of phenotypic variation of hybridized populations in new environments suggests that the genetic influence of hatchery fish on wild Algonquin populations may have been negated during the 5–11 generations that followed the most recent hybridization event. The number of generations required for selection to remove maladaptive domestic alleles from wild populations is still unknown. The elevated survival of the more recently stocked mildly hybridized population may suggest that after approximately 5 generations, some effect of these alleles is still present. Yet the lack of support for added plasticity or phenotypic variance in this same population suggests that either the effect of such residual alleles is minimal after approximately 5 generations or that something else may be responsible for the increased survival of this population in the two study lakes in question. What this may be is as yet unknown, but the supplementary analysis on habitat dissimilarity suggests it is not the result of the mildly hybridized population experiencing less selective pressures in the new environments than other populations. Maternal effects may be responsible as the mildly hybridized population were significantly larger than the other three populations 41 days prior to stocking; however, the range in mean size between the largest and smallest (hatchery) populations (2.22 mm) decreased to 0.8 mm 6 days prior to stocking, with the position of these two populations reversing (data not shown). Additionally, no fewer than 11 females were used to create the experimental crosses, minimizing the chances of a single female affecting the results.

Our meta-analysis results, while confirming that domesticated-wild hybrids almost always have reduced survival relative to wild fish in their local environment (see also Araki et al. 2007), also suggest that domestic genes, among the early generations of hybrids, provide hybridized populations with a fitness advantage in new environments. We therefore think it is reasonable to assume that while the fitness of stocked populations in Algonquin Park was reduced following hybridization, these same populations would have experienced a fitness advantage relative to nonstocked populations if they had been exposed to novel environmental change. The lack of evidence for that now, 5–11 generations later, suggests that selection has removed any detectable effect of hybridization on the adaptive potential of hybrid populations.

### Possible caveats and alternative explanations

Although we believe that our study provides evidence that selection, over time, removes the adaptive advantages that hybridized populations should experience as a result of increased genetic diversity, the exact nature of the hybridization events that took place in the two hybridized populations is unknown. For example, while more foreign genetic material might be expected to remain in the more recently stocked mildly hybridized population, perhaps none of the relatively few hatchery fish stocked into this population in 1994 survived to adulthood and successfully reproduced. In which case, current admixture levels of this population are the result of earlier hybridization events. If this is true, then the increased survival of the mildly hybridized population in two of the three lakes is the result of foreign genes persisting longer than expected or some other untested factor such as maternal effects. To address the question of whether the hybridized populations possess more additive genetic variation directly, loci in coding regions of the genome require examination to determine whether genetic diversity at quantitative trait loci is actually still greater in hybridized populations after multiple generations of selection.

In addition, 16–32 years have passed since stocking occurred in the hybridized populations. Contemporary hatchery fish might therefore differ from their progenitors in terms of phenotype, adaptability or survival in new environments. Nevertheless, genetic drift and directional selection have likely been minimal over this period because the Hills Lake strain has always been maintained with a high effective population size and had been in the hatchery environment for 10+ generations prior to being stocked in our hybridized lakes (Fraser 1981). Indeed, previous studies have found that the rate of wild fitness loss experienced by salmonids introduced to the hatchery environment is greatest in the first few generations of domestication and tapers off after four or five generations (Araki et al. 2007).

### Conclusions and future directions

Improved knowledge of the long-term fitness outcomes of hybridized populations induced by human activities will be important for sorting out conservation issues associated with hybrids. Some issues, such as the lack of knowledge about adaptive potential in the hybridized population before hybridization, may be difficult to address. Other issues, such as whether hybridized populations experience a loss of local adaptation to environmental extremes, may be tested in controlled experimental settings. Addressing these concerns could potentially explain in what situations the negative effects of hybridization will persist over time and in what situations they will not.

Although we believe that our study demonstrates that salmonid populations can exhibit no effect of hybridization after 5–11 generations, more studies of this phenomenon are still required to aid policy makers when classifying the protection status or management practices for populations known to be hybridized. Our results additionally provide hope for wild populations of high ecological and economical value currently displaying negative effects as a result of human-mediated hybridization with domesticated conspecifics. If the incoming flow of foreign genes can be stemmed and the environment resembles that experienced by the wild population prior to hybridization, there appears to be a considerable chance that populations will recover, and possibly in less time than previously thought. Similar conclusions have recently been made about canid species exposed to hybridization, but that continue to experience the same selective regimes of their nonhybridized ancestors (Stronen and Paquet 2013).

A final conservation consideration that may be drawn from our work relates to domesticated populations that have become naturalized. When occurring sympatrically with wild conspecifics, these are often seen as a problem due to the high potential of hybridization. Yet the conservation value of such populations might often be underestimated in situations where they occur in neighbouring habitats to wild populations or have entered the environment following the extirpation of their wild counterparts. Certainly, domesticated populations are genetically exotic. As our study enforces, however, natural selection may shift phenotypic and fitness to levels similar to those exhibited by wild populations in environments identical or similar to those experienced by wild conspecifics, and so naturalized, domesticated populations can come to play the same ecological role as a wild population.

## Data archiving statement

Data for this study are available from the Dryad Digital Repository: http://doi.org/10.5061/dryad.ns905.

## Supporting Information

**Appendix S1.** Length and weight information for brook trout fry prior to stocking. **Appendix S2.** Supplementary analysis of bias introduced by sampling procedure. **Appendix S3.** Location of landmarks used for morphology analysis. **Appendix S4.** Morphological and survival differences: Tukey's Honest Significant Differences (*P*-value ranges include *P* > 0.05 (−), *P* < 0.05 (*), *P* < 0.01 (**), and *P* < 0.001 (***)) were calculated for the four experimental populations: Wild-Nonstocked (W), Mildly-Hybridized (MH), Highly-Hybridized (HH), and Hatchery (H) in the three transplant lakes, stocked May 15th 2010. **Appendix S5.** Results of the GLMs implemented in R using the quasi-Poisson and Gaussian error distributions to detect interaction among study lakes (Lake) and source populations (Source).

**Appendix S6.** Summary of studies incorporated in a meta-analysis on survival of domestic-wild hybrids and wild salmonids in natural settings.
