# Supplementary material for: Does human-induced hybridization have long-term genetic effects? Empirical testing with domesticated, wild and hybridized fish populations
Source: Evol Appl. 2014 Aug 27;7(10):1180–91. doi: 10.1111/eva.12199 (PMC4275090; doi:10.1111/eva.12199)
Supplement: Supplementary file 1 [file eva0007-1180-sd1.docx]

**Appendix S1: Length and weight information for brook trout fry prior to stocking**

Lengths (mm) of fry from three source populations in Algonquin Park and a fourth hatchery strain used for stocking the two hybridized population previously. Measurements were taken on April 4^th^ 2010, 41 days prior to stocking into three study lakes north of Lake Huron.

| **Sample** | **Wild-Nonstocked** | **Mildly-Hybridized** | **Highly-Hybridized** | **Hatchery** |
| --- | --- | --- | --- | --- |
| 1 | 25.35 | 28.64 | 25.32 | 24.87 |
| 2 | 27.05 | 25.64 | 23.37 | 25.08 |
| 3 | 24.47 | 24.33 | 25.19 | 25.49 |
| 4 | 25.78 | 24.82 | 24.33 | 21.72 |
| 5 | 25.09 | 28.34 | 26.52 | 25.18 |
| 6 | 26.19 | 26.93 | 23.90 | 26.21 |
| 7 | 26.24 | 23.61 | 23.11 | 24.77 |
| 8 | 26.29 | 28.85 | 25.75 | 24.27 |
| 9 | 23.43 | 27.04 | 24.95 | 26.39 |
| 10 | 26.97 | 23.15 | 25.91 | 23.77 |
| 11 | 25.10 | 24.04 | 28.43 | 20.98 |
| 12 | 25.78 | 28.68 | 25.19 | 25.50 |
| 13 | 25.49 | 27.17 | 26.70 | 25.29 |
| 14 | 24.85 | 24.19 | 26.82 | 24.49 |
| 15 | 24.57 | 28.04 | 24.00 | 27.74 |
| 16 | 27.48 | 29.98 | 23.80 | 23.21 |
| 17 | 24.26 | 28.13 | 25.30 | 26.65 |
| 18 | 25.08 | 26.64 | 27.82 | 20.22 |
| 19 | 24.16 | 26.80 | 25.82 | 24.26 |
| 20 | 23.20 | 28.11 | 25.93 | 22.66 |
| **Mean** | 25.34 | 26.66 | 25.41 | 24.44 |
| **SE** | 0.26 | 0.45 | 0.32 | 0.43 |

Mean weight (g) of fry from three source populations in Algonquin Park and a fourth hatchery strain used for stocking the two hybridized population previously. Measurements were taken on April 4^th^ 2010, 41 days prior to stocking into three study lakes north of Lake Huron. Numbers in brackets are the number of fry used to calculate means.

| **Weighing** | **Wild-nonstocked** | **Mildly-Hybridized** | **Highly-Hybridized** | **Hatchery** |
| --- | --- | --- | --- | --- |
| 1st Mean | 0.114(251) | 0.125(226) | 0.110(262) | 0.103(392) |
| 2nd Mean | 0.113(257) | 0.133(229) | 0.112(273) | 0.109(218) |
| 3rd Mean | 0.115(234) | 0.130(175) | 0.111(272) | 0.112(212) |
| Grand Mean | 0.114 | 0.125 | 0.111 | 0.108 |

**Appendix S2: Supplementary analysis of bias introduced by sampling procedure.**

As the duration of each net set and the number of net sets each lake received were not consistent (longer soak times and a supplemental sampling were used to boost catches when returns were low) it was necessary to insure that these discrepancies did not introduce bias towards any one source population. Tests were conducted to insure unbiased results separately for these two areas of potential concern.

To test for bias imposed by longer or shorter capture events analyses of variance (ANOVAs) using a quasi-Poisson error distribution were conducted on the proportions of the total catch per net set made up by each source population in each study lake against the duration of the capture event (< 1hr, short; < 4 hr, medium; > 4hr, long). Models were compared to null models, containing only the intercept, using Akaike’s Information Criterion (AIC). The results supported the null hypothesis, indicating that the proportions of the total catch made up by each source population were consistent regardless of the duration of the netting event (with one exception in 12 tests, the hybridized population in Lake A made up a larger proportion of the catches following longer net sets).

To insure that a greater number of sampling days did not introduce bias, catches of each source population modeled in a GLM using every two way interaction among source population, study lake and the chronological series of net sets using a quasi-Poisson distribution. The results indicated that more fish were captured later on in the experiment, however there was no significant interactions between the number of chronological sequence of net sets and the source populations captured.

The results of these supplemental tests suggest that we are justified in inferring that any differences in the abundances of source populations captured in a particular net set represent actual differences in abundance within the study lakes, rather than differences due to sampling procedure.**Appendix S3: Location of landmarks used for morphology analysis**


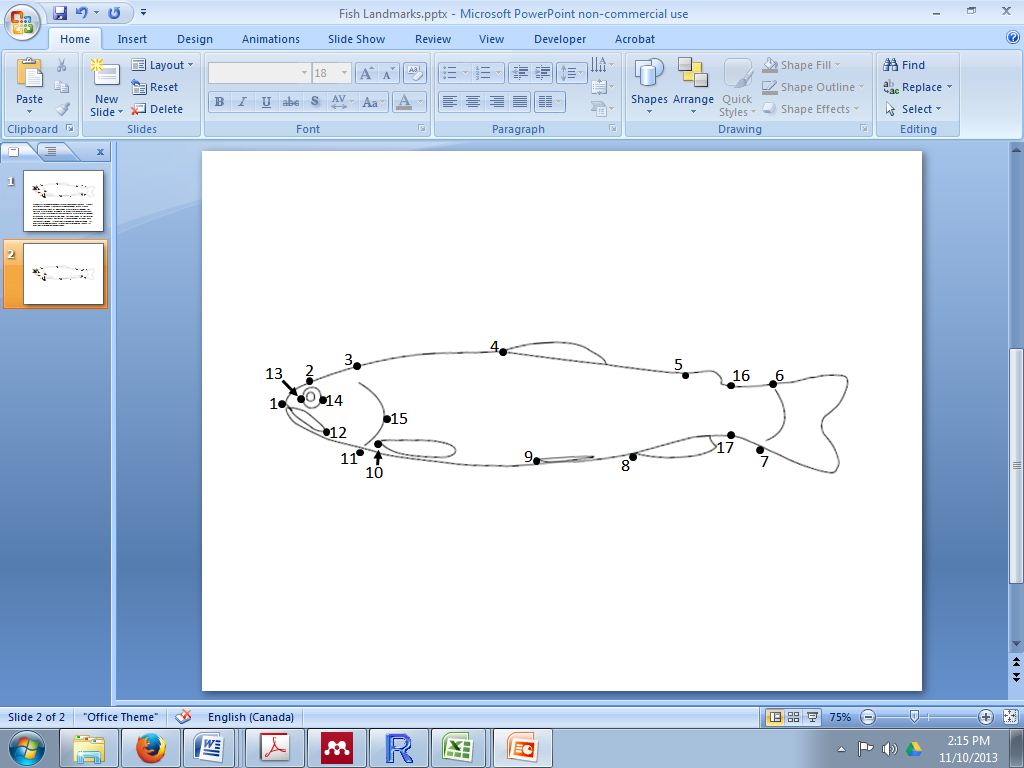


Landmarks for geomorphometric analysis on brook trout: 1, the most anterior part of body; 2, the head directly above midpoint of the eye; 3, the head directly above dorsal limit of operculum; 4, the anterior insertion point for dorsal fin; 5, the anterior limit of adipose fin; 6, the dorsal terminus of the caudal peduncle; 7, the ventral terminus of the caudal peduncle; 8, the anterior insertion point of the anal fin; 9, the anterior insertion point for the left pelvic fin; 10, the anterior insertion point for the left pectoral fin; 11, the meeting point of the gill plate and the ventral midline; 12, the most posterior point on upper mandible; 13, the most anterior point on the eye; 14, the most posterior point on the eye; 15, the most posterior point on the operculum; 16, the dorsal position above the thinnest part of the caudal peduncle; 17, the ventral position below the thinnest part of the caudal peduncle.

Appendix S4. Morphological and survival differences: Tukey’s Honest Significant Differences (P-value ranges include p > 0.05 (-), p < 0.05 (*), p < 0.01 (**), and p < 0.001 (***)) were calculated for the four experimental populations: Wild-Nonstocked (W), Mildly-Hybridized (MH), Highly-Hybridized (HH), and Hatchery (H) in the three transplant lakes, stocked May 15^th^ 2010.

| Variable | Experimental Lakes | | | | | | | | | | | |
| --- | --- | --- | --- | --- | --- | --- | --- | --- | --- | --- | --- | --- |
|  |  | Lake A | | |  | Lake B | | |  | Lake C | | |
| Survival |  | W | MH | HH |  | W | MH | HH |  | W | MH | HH |
|  | MH | *** |  |  | MH | *** |  |  | MH | - |  |  |
|  | HH | - | *** |  | HH | - | *** |  | HH | - | - |  |
|  | H | - | *** | - | H | - | *** | - | H | *** | ** | ** |
|  |  | Lake A | | |  | Lake B | | |  | Lake C | | |
| Centroid |  | W | MH | HH |  | W | MH | HH |  | W | MH | HH |
|  | MH | - |  |  | MH | - |  |  | MH | *** |  |  |
|  | HH | - | - |  | HH | - | - |  | HH | ** | - |  |
|  | H | ** | ** | ** | H | - | - | - | H | *** | * | ** |
|  |  | Lake A | | |  | Lake B | | |  | Lake C | | |
| RW1 |  | W | MH | HH |  | W | MH | HH |  | W | MH | HH |
|  | MH | - |  |  | MH | - |  |  | MH | - |  |  |
|  | HH | - | - |  | HH | - | - |  | HH | - | - |  |
|  | H | - | *** | - | H | - | - | - | H | - | - | - |
|  |  | Lake A | | |  | Lake B | | |  | Lake C | | |
| RW3 |  | W | MH | HH |  | W | MH | HH |  | W | MH | HH |
|  | MH | * |  |  | MH | - |  |  | MH | _ |  |  |
|  | HH | - | - |  | HH | - | - |  | HH | - | - |  |
|  | H | ** | *** | ** | H | - | * | ** | H | ** | *** | *** |
|  |  | Lake A | | |  | Lake B | | |  | Lake C | | |
| RW4 |  | W | MH | HH |  | W | MH | HH |  | W | MH | HH |
|  | MH | *** |  |  | MH | - |  |  | MH | *** |  |  |
|  | HH | ** | - |  | HH | - | - |  | HH | *** | - |  |
|  | H | *** | *** | *** | H | *** | *** | * | H | *** | *** | ** |

**Appendix S5.** Results of the GLMs implemented in R using the quasi-Poisson and Gaussian error distributions to detect interaction among study lakes (Lake) and source populations (Source)

**Model Selection Tables:**

*Centroid Size*

| Levels | Intercept | Lake | Source | Lake X Source | d.f. | logLikelihood | AIC | deltaAIC |
| --- | --- | --- | --- | --- | --- | --- | --- | --- |
| 8 | 19.16 | + | + | + | 13 | -565.99 | 1158.00 | 0.00 |
| 4 | 19.40 | + | + |  | 7 | -574.08 | 1162.20 | 4.18 |
| 2 | 19.19 | + |  |  | 4 | -591.43 | 1190.90 | 32.88 |
| 3 | 20.34 |  | + |  | 5 | -667.09 | 1344.20 | 186.19 |
| 1 | 20.92 |  |  |  | 2 | -671.40 | 1346.80 | 188.81 |

*Relative Warp 1*

| Levels | Intercept | Centroid | Lake | Source | Lake X Source | df | logLikelihood | AIC | delta |
| --- | --- | --- | --- | --- | --- | --- | --- | --- | --- |
| 8 | -0.0506 | 1.94E-03 | + | + |  | 8 | 855.94 | -1695.90 | 0.00 |
| 16 | -0.0510 | 1.95E-03 | + | + | + | 14 | 860.79 | -1693.60 | 2.31 |
| 4 | -0.0461 | 1.76E-03 | + |  |  | 5 | 848.38 | -1686.80 | 9.12 |
| 3 | -0.0123 |  | + |  |  | 4 | 795.68 | -1583.40 | 112.53 |
| 15 | -0.0137 |  | + | + | + | 13 | 804.15 | -1582.30 | 113.58 |
| 7 | -0.0128 |  | + | + |  | 7 | 798.14 | -1582.30 | 113.61 |
| 6 | -0.0527 | 2.36E-03 |  | + |  | 6 | 786.85 | -1561.70 | 134.19 |
| 2 | -0.0520 | 2.40E-03 |  |  |  | 3 | 777.57 | -1549.10 | 146.75 |
| 5 | -0.0046 |  |  | + |  | 5 | 696.89 | -1383.80 | 312.11 |
| 1 | -0.0017 |  |  |  |  | 2 | 688.03 | -1372.10 | 323.82 |

*Relative warp 3*

| Levels | Intercept | Centroid | Lake | Source | Lake X Source | d.f. | logLikelihood | AIC | deltaAIC |
| --- | --- | --- | --- | --- | --- | --- | --- | --- | --- |
| 16 | 0.0038 | -3.80E-04 | + | + | + | 14 | 835.90 | -1643.80 | 0.00 |
| 8 | 0.0026 | -2.96E-04 | + | + |  | 8 | 828.99 | -1642.00 | 1.83 |
| 15 | -0.0034 |  | + | + | + | 13 | 833.68 | -1641.40 | 2.45 |
| 7 | -0.0031 |  | + | + |  | 7 | 827.62 | -1641.20 | 2.57 |
| 6 | 0.0021 | -1.98E-04 |  | + |  | 6 | 824.73 | -1637.50 | 6.35 |
| 5 | -0.0019 |  |  | + |  | 5 | 823.44 | -1636.90 | 6.92 |
| 3 | -0.0018 |  | + |  |  | 4 | 809.22 | -1610.40 | 33.36 |
| 4 | -0.0003 | -7.79E-05 | + |  |  | 5 | 809.32 | -1608.60 | 35.17 |
| 1 | -0.0007 |  |  |  |  | 2 | 805.95 | -1607.90 | 35.90 |
| 2 | 0.0011 | -8.78E-05 |  |  |  | 3 | 806.18 | -1606.40 | 37.45 |

*Relative warp 4*

| Levels | Intercept | Centroid | Lake | Source | Lake X Source | d.f. | logLikelihood | AIC | deltaAIC |
| --- | --- | --- | --- | --- | --- | --- | --- | --- | --- |
| 8 | 0.0128 | -7.30E-04 | + | + |  | 8 | 901.51 | -1787.00 | 0.00 |
| 6 | 0.0074 | -4.31E-04 |  | + |  | 6 | 896.71 | -1781.40 | 5.62 |
| 16 | 0.0127 | -7.37E-04 | + | + | + | 14 | 903.09 | -1778.20 | 8.86 |
| 5 | -0.0014 |  |  | + |  | 5 | 885.93 | -1761.90 | 25.17 |
| 7 | -0.0014 |  | + | + |  | 7 | 887.02 | -1760.00 | 26.99 |
| 15 | -0.0014 |  | + | + | + | 13 | 889.10 | -1752.20 | 34.82 |
| 4 | 0.0216 | -1.18E-03 | + |  |  | 5 | 860.99 | -1712.00 | 75.06 |
| 2 | 0.0093 | -4.65E-04 |  |  |  | 3 | 838.33 | -1670.70 | 116.37 |
| 1 | -0.0005 |  |  |  |  | 2 | 830.29 | -1656.60 | 130.44 |
| 3 | -0.0010 |  | + |  |  | 4 | 831.72 | -1655.40 | 131.58 |
