# Supplementary material for: Does human-induced hybridization have long-term genetic effects? Empirical testing with domesticated, wild and hybridized fish populations
Source: Evol Appl. 2014 Aug 27;7(10):1180–91. doi: 10.1111/eva.12199 (PMC4275090; doi:10.1111/eva.12199)
Supplement: Supplementary file 2 — Appendix S6. Summary of studies incorporated in a meta-analysis on survival of domestic-wild hybrids and wild salmonids in natural settings. [file eva0007-1180-sd2.docx]

**Appendix S6.** Summary of studies incorporated in a meta-analysis on survival of domestic-wild hybrids and wild salmonids in natural settings.

| **Species** | **Life-stages stocked and study period** | **Wild Status** | | **Hatchery Status** | | **N**** | **Hybrid Generation** | **References** |
| --- | --- | --- | --- | --- | --- | --- | --- | --- |
|  |  | **Local** | **Foreign** | **Local** | **Non-Local** |  |  |  |
| Salvelinus fontinalis | 0+ Fry, Multi-year | - | 6 | - | 6 | 6 | F1 | Gowing (1986) |
| Salmo salar | Eyed Eggs, Outmigration of parr/smolt | 3 | - | - | 3 | 3 | F2, BC | McGinnity et al. (2003) |
| Oncorhynchus mykiss | 0+ Fry, 1-2 Summers | 8 | - | 8 | - | 8 | F1 | Miller, Close, & Kapuscinski (2004) |
| Salvelinus fontinalis | Eyed Eggs/Unfed Fry, 1 year | 4 | - | 4 | - | 4 | F1 | Reisenbichler and McIntyre (1977) |
| Salmo salar | Eyed Eggs, Multi-year | - | 3 | - | 3 | 3 | F1* | Skaala et al. (2012) |
| Oncorhynchus mykiss | 0+ Fry, 1 Summer | - | 6 | - | 6 | 6 | F1, BC1, BC2 | Vandersteen et al. (2012) |
| Salvelinus fontinalis | 0+ Fry, 1 Summer | 1 | 7 | - | 8 | 8 | F1 | Webster and Flick (1981) |

* not explicitly stated

** the number of comparisons incorporated in this study

**Literature Cited:**

Gowing, H. 1986. Survival and Growth of Matched Plantings of Assinica Strain Brook Trout and Hybrid Brook Trout (Assinica Male X Domestic Female) in Six Small Michigan Lakes. North American Journal of Fisheries Management 6:242–251.

McGinnity, P., P. Prodöhl, A. Ferguson, R. Hynes, N. O. Maoiléidigh, N. Baker, D. Cotter, B. O’Hea, D. Cooke, G. Rogan, J. Taggart, and T. Cross. 2003. Fitness reduction and potential extinction of wild populations of Atlantic salmon, Salmo salar, as a result of interactions with escaped farm salmon. Proceedings. Biological sciences / The Royal Society 270:2443–50.

Miller, L. M., T. Close, and A. R. Kapuscinski. 2004. Lower fitness of hatchery and hybrid rainbow trout compared to naturalized populations in Lake Superior tributaries. Molecular ecology 13:3379–88.

Reisenbichler, R. R., and J. D. McIntyre. 1977. Genetic Differences in Growth and Survival of Juvenile Hatchery and Wild Steelhead Trout, Salmo gairdneri. Journal of the Fisheries Research Board of Canada 34:123–128.

Skaala, Ø., K. A. Glover, B. T. Barlaup, T. Svåsand, F. Besnier, M. M. Hansen, R. Borgstrøm, and I. A. Fleming. 2012. Performance of farmed, hybrid, and wild Atlantic salmon ( Salmo salar ) families in a natural river environment. Canadian Journal of Fisheries and Aquatic Sciences 69:1994–2006.

Vandersteen, W., P. Biro, L. Harris, and R. Devlin. 2012. Introgression of domesticated alleles into a wild trout genotype and the impact on seasonal survival in natural lakes. Evolutionary Applications 5:76–88.

Webster, D. A., and W. A. Flick. 1981. Performance of Indigenous, Exotic, and Hybrid Strains of Brook Trout ( Salvelinus fontinalis ) in Waters of the Adirondack Mountains, New York. Canadian Journal of Fisheries and Aquatic Sciences 38:1701–1707.
